# Generalized Centroid Estimators in Bioinformatics

**DOI:** 10.1371/journal.pone.0016450

**Published:** 2011-02-18

**Authors:** Michiaki Hamada, Hisanori Kiryu, Wataru Iwasaki, Kiyoshi Asai

**Affiliations:** 1 Graduate School of Frontier Sciences, The University of Tokyo, Chiba, Japan; 2 Computational Biology Research Center, National Institute of Advanced Industrial Science and Technology (AIST), Tokyo, Japan; Memorial Sloan Kettering Cancer Center, United States of America

## Abstract

In a number of estimation problems in bioinformatics, accuracy measures of the target problem are usually given, and it is important to design estimators that are suitable to those accuracy measures. However, there is often a discrepancy between an employed estimator and a given accuracy measure of the problem. In this study, we introduce a general class of efficient estimators for estimation problems on high-dimensional binary spaces, which represent many fundamental problems in bioinformatics. Theoretical analysis reveals that the proposed estimators generally fit with commonly-used accuracy measures (e.g. sensitivity, PPV, MCC and F-score) as well as it can be computed efficiently in many cases, and cover a wide range of problems in bioinformatics from the viewpoint of the principle of maximum expected accuracy (MEA). It is also shown that some important algorithms in bioinformatics can be interpreted in a unified manner. Not only the concept presented in this paper gives a useful framework to design MEA-based estimators but also it is highly extendable and sheds new light on many problems in bioinformatics.

## Introduction

In estimation problems in bioinformatics, the space of solutions is generally large and often high-dimensional. Among them, a number of fundamental problems in bioinformatics, such as alignment of biological sequences, prediction of secondary structures of RNA sequences, prediction of biological networks, and estimation of phylogenetic trees, are classified into estimation problems whose solutions are in a high-dimensional binary space. Such problems are generally difficult to solve, and the estimates are often unreliable.

The popular solutions for these problems, such as for the secondary structure of RNA with minimum free energy, are the maximum likelihood (ML) estimators. The ML estimator maximizes the probability that the estimator is exactly correct, but that probability is generally very small. Noticing the drawbacks of the ML estimators, Carvalho and Lawrence have proposed the *centroid estimator*, which represents an ensemble of all the possible solutions and minimizes the expected Hamming loss of the prediction [1].

In this paper, we conduct a theoretical analysis of estimation problems in high-dimensional binary space, and present examples and solutions in bioinformatics. The theories in this paper provide a unified framework for designing superior estimators for estimation problems in bioinformatics. The estimators discussed in this paper, including the ML estimator and the centroid estimator, are formalized as maximum expected gain (MEG) estimators, which maximize the estimator-specific gain functions with respect to the given probability distribution. The objective of the estimation is not always to find the exact solution with an extremely small probability or to find the solution with the minimum Hamming loss, but rather to find the most accurate estimator. Therefore, we adopt the principle of maximum expected accuracy (MEA), which has been successfully applied to various problems in bioinformatics, such as the alignment of biological sequences [2]–[4], the secondary structure prediction of RNA [5]–[8] and other applications [9]–[11].

Theoretical analysis, however, shows that those MEA estimators are not always robust with respect to accuracy measures. To address this, we previously proposed the 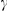-centroid estimator in a few specific problems [4], [12]. In this paper, in order to make the 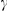-centroid estimator easily applicable to other estimation problems, we introduce an abstract form of the 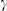-centroid estimator, which is defined on general binary spaces and designed to fit to the commonly used accuracy measures. The 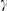-centroid estimator is a generalization of the centroid estimator, and offers a more robust framework for estimators than the previous estimators. We extend the theory of maximum expected gain (MEG) estimators and 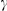-centroid estimators for two advanced problems: the estimators that represent the common solutions for multiple entries, and the estimators for marginalized probability distributions.

## Materials and Methods

**Problem 1 (Pairwise alignment of two biological sequences)** *Given a pair of biological (DNA, RNA, protein) sequences* 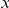 *and* 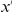 *, predict their alignment as a point in* 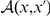 *, the space of all the possible alignments of* 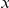 *and* 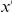 *.*

**Problem 2 (Prediction of secondary structures of RNA sequences)** *Given an RNA sequence* 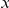 *, predict its secondary structure as a point in* 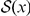 *, the space of all the possible secondary structures of* 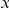 *.*

A point in 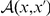, can be represented as a binary vector of 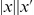 dimensions by denoting the aligned bases across the two sequences as “1” and the remaining pairs of bases as “0”. A point in 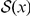 can also be represented as a binary vector of 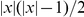 dimensions, which represent all the pairs of the base positions in 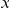, by denoting the base pairs in the secondary structures as “1”. In each problem, the predictive space (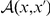 or 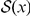) is a subset of a binary space (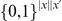 or 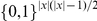) because the combinations of aligned bases or base pairs are restricted (see “Discrete (binary) spaces in bioinformatics” in Appendices for more formal definitions). Therefore, Problem 1 and Problem 2 are special cases of the following more general problem:

**Problem 3 (Estimation problem on a binary space)** *Given a data set* 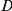 *and a predictive space* 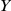 *(a set of all candidates of a prediction), which is a subset of* 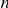 *-dimensional binary vectors* 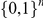 *, that is,* 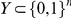 *, predict a point* 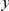 *in the predictive space* 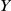 *.*

Not only Problem 1 and Problem 2 but also a number of other problems in bioinformatics are formulated as Problem 3, including the prediction of biological networks and the estimation of phylogenetic trees (Problem 4).

To discuss the stochastic character of the estimators, the following assumption is introduced.

**Assumption 1 (Existence of probability distribution)** *In Problem 3, there exists a probability distribution* 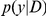 *on the predictive space* 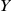 *.*

For Problem 3 with Assumption 1, we have the following Bayesian maximum likelihood (ML) estimator.

**Definition 1 (Bayesian ML estimator** [**1**] **)** *For Problem 3 with Assumption 1, the estimator*

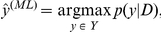

*which maximizes the Bayesian posterior probability* 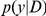 *, is referred to as a Bayesian maximum likelihood (ML) estimator.*

For problems classified as Problem 3, Bayesian ML estimators have dominated the field of estimators in bioinformatics for years. The classical solutions of Problem 1 and Problem 2 are regarded as Bayesian ML estimators with specific probability distributions, as seen in the following examples.

**Example 1 (Pairwise alignment with maximum score)** *In Problem 1 with a scoring model (e.g., gap costs and a substitution matrix), the distribution* 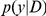 *in Assumption 1 is derived from the Miyazawa model* [*13*] *(See “Probability distributions* 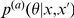 on 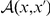
”* in* *Appendices* *), and the Bayesian ML estimator is equivalent to the alignment that has the highest similarity score.*

**Example 2 (RNA structure with minimum free energy)** *In Problem 2 with a McCaskill energy model* [*14*] *, the distribution* 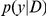 *in Assumption 1 can be obtained with the aid of thermodynamics (See “Probability distributions* 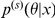 *on* 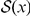 *” in* *Appendices* *for details), and the Bayesian ML estimator is equivalent to the secondary structure that has the minimum free energy (MFE).*

When a stochastic model such as a pair hidden Markov model (pair HMM) in Problem 1 or a stochastic context-free grammar (SCFG) in Problem 2 is assumed in such problems, the distribution and the ML estimator are derived in a more direct manner.

The Bayesian ML estimator regards the solution which has the highest probability as the most likely one. To provide more general criteria for good estimators, here we define the *gain function* that gives the gain for the prediction, and the *maximum expected gain (MEG) estimator* that maximizes the *expected gain*.

**Definition 2 (Gain function)** *In Problem 3, for a point* 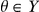 *and its prediction* 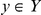 *, a gain function is defined as* 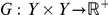 *,* 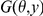 *.*

**Definition 3 (MEG estimator)** *In Problem 3 with Assumption 1, the maximum expected gain (MEG) estimator is defined as*

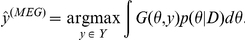



If the gain function is designed according to the *accuracy measures* of the target problem, the MEG estimator is considered as the maximum expected accuracy (MEA) estimator, which has been successfully applied in bioinformatics (e.g., [9]).Although in estimation theory a *loss function* that should be minimized is often used, in order to facilitate the understanding of the relationship with the MEA, in this paper, we use a *gain function* that should be maximized.

The MEG estimator for the gain function 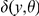 is the ML estimator. Although this means that the ML estimator maximizes the probability that the estimator is identical to the true value, there is an extensive collection of suboptimal solutions and the probability of the ML estimator is extremely small in cases where 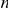 in Problem 3 is large. Against this background, Carvalho and Lawrence proposed the *centroid estimator*, which takes into account the overall ensemble of solutions [1]. The centroid estimator can be defined as an MEG estimator for a *pointwise gain function* as follows:

**Definition 4 (Pointwise gain function)** *In Problem 3, for a point* 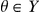 *and its prediction* 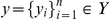 *, a gain function* 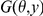 *written as*

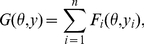
(1)
*where* 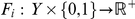 *(* 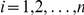 *), is referred to as a pointwise gain function.*

**Definition 5 (Centroid estimator** [**1**] **)** *In Problem 3 with Assumption 1, a centroid estimator is defined as an MEG estimator for the pointwise gain function given in Eq.* *(1)* *by defining* 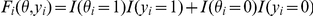 *.*

Throughout this paper, 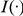 is the indicator function that takes a value of 1 or 0 depending on whether the condition constituting its argument is true or false. The centroid estimator is equivalent to the expected Hamming loss minimizer [1]. If we can maximize the pointwise gain function independently in each dimension, we can obtain the following *consensus estimator*, which can be easily computed.

**Definition 6 (Consensus estimator** [**1**] **)** *In Problem 3 with Assumption 1, the* consensus estimator 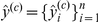 *for a pointwise gain function is defined as*

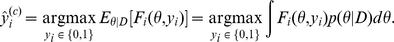



The consensus estimator is generally *not* contained within the predictive space 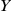 since the predictive space 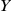 usually has complex constraints for each dimension (see “Discrete (binary) spaces in bioinformatics” in Appendices). Carvalho and Lawrence proved a sufficient condition for the centroid estimator to contain the consensus estimator (Theorem 2 in [1]). Here, we present a more general result, namely, a sufficient condition for the MEG estimator for a pointwise function to contain the consensus estimator.

**Theorem 1** *In Problem 3 with Assumption 1 and a pointwise gain function, let us suppose that a predictive space* 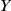 *can be written as*

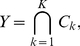
(2)where 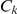 *is defined as *

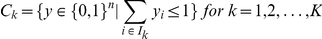

*for an index-set* 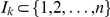 *. If the pointwise gain function in Eq.* *(1)* *satisfies the condition*

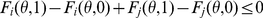
(3)
*for every* 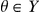 *and every* 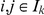 *(* 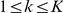 *), then the consensus estimator is in the predictive space* 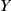 *, and hence the MEG estimator contains the consensus estimator.*

The above conditions are frequently satisfied in bioinformatics problems (see Appendices for examples).

## Results

### 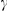-centroid estimator: generalized centroid estimator

In Problem 3, the “1”s and the “0”s in the binary vector of a prediction 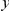 can be interpreted as positive and negative predictions, respectively. The respective numbers of true positives (TP), true negatives (TN), false positives (FP) and false negatives (FN) for a point 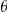 and its prediction 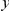 are denoted by 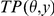, 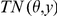, 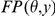 and 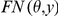, respectively (See also Eqs (15)–(18)).

To design a *superior* MEG estimator, it is natural to use a gain function of the following form, which yields positive scores for the number of true predictions (TP and TN) and negative scores for those of false predictions (FP and FN):
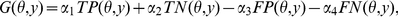
(4)where 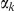 is a positive constant (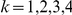). Note that this gain function is a pointwise gain function.

This gain function is naturally compatible with commonly used accuracy measures such as sensitivity, PPV, MCC and F-score (a function of TP, TN, FP and FN; see “Evaluation measures defined using TP, TN, FP and FN” in Appendices for definitions). The following Definition 7 and Theorem 2 characterize the MEG estimator for this gain function.

**Definition 7 (** 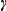 **-centroid estimator)** *In Problem 3 with Assumption 1 and a fixed* 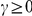 *, the* 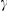 *-centroid estimator is defined as the MEG estimator for the pointwise gain function given in* *Eq. (1)* *by *

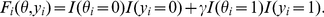
(5)


**Theorem 2** *The MEG estimator for the gain function in* *Eq. (4)* *is equivalent to a* 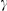 *-centroid estimator with* 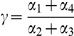 *.*

Theorem 2 (see Appendices for a formal proof) is derived from the following relations:
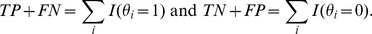



The 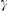-centroid estimator maximizes the expected value of 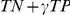, and includes the centroid estimator as a special case where 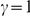. The parameter 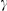 adjusts the balance between the gain from true negatives and that from true positives.

The expected value of the gain function of the 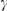-centroid estimator is computed as follows (see Appendices for the derivation):
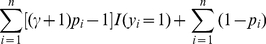
(6)where
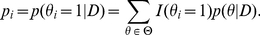
(7)


Since the second term in Eq. (6) does not depend on 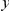, the 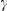-centroid estimator maximizes the first term. The following theorem is obtained by assuming the additional condition described below.

**Theorem 3** *In Problem 3 with Assumption 1, the predictive space* 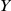 *satisfies the following condition: if* 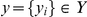 *, then* 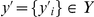 *where* 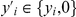 *for all* 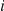 *. Then, the* 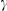 *-centroid estimator is equivalent to the estimator that maximizes the sum of marginalized probabilities* 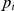 *that are greater than* 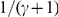 *in the prediction.*

The condition is necessary to obtain 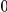 for the 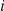 that produces negative values in the first term in Eq. (6). Problem 2, Problem 1, and many other typical problems in bioinformatics satisfy this condition. Because the pointwise gain function of the 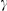-centroid estimator satisfies Eq. (3) in Theorem 1, we can prove the following Corollary 1.

**Corollary 1 (** 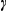 **-centroid estimator for** 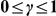 **)** *In Problem 3 with Assumption 1, the predictive space* 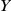 *is given in the same form in* *Eq. (2)* *of Theorem 1. Then, the* 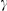 *-centroid estimator for* 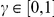 *contains its consensus estimator. Moreover, the consensus estimator is identical to the following estimator* 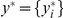 *:*

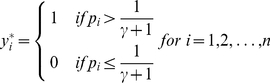
(8)
*where* 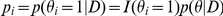 *.*

Here, 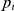 is the marginalized probability of the distribution for the 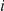-th dimension of the predictive space. In Problem 1, it is known as the alignment probability, which is defined as the probability of each pair of positions across the two sequences being aligned. In Problem 2, it is known as the base pairing probability, which is defined as the probability of each pair of positions forming a base pair in the secondary structure. These marginalized probabilities can be calculated by using dynamic programming algorithms, such as the forward-backward algorithm and the McCaskill algorithm, depending on the model of the distributions. (see “Probability distributions on discrete spaces” in Appendices for those distributions).

Corollary 1 does not hold for 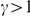, but in typical problems in bioinformatics the 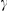-centroid estimator for 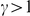 can be calculated efficiently by using dynamic programming, as shown in the following examples.

**Example 3 (** 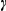 **-centroid estimator of pairwise alignment)** *In Problem 1 with Assumption 1, the* 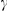 *-centroid estimator maximizes the sum of the alignment probabilities which are greater than* 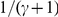 *(Theorem 3), and for* 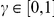 *it can be given as the consensus estimator calculated from* *Eq. (8)* *(Corollary 1). For* 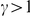 *, the* 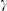 *-centroid estimator is obtained by using a dynamic programming algorithm with the same type of iterations as in the Needleman-Wunsch algorithm:*

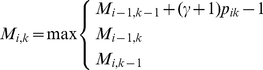
(9)
*where* 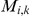 *stores the optimal value of the alignment between two sub-sequences,* 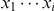 *and* 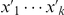 *(see “Secondary structure prediction of an RNA sequence (Problem 2)” in* *Appendices* *for detailed descriptions).*

**Example 4 (** 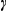 **-centroid estimator for prediction of secondary structures)** *In Problem 2 with Assumption 1, the* 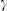 *-centroid estimator maximizes the sum of the base pairing probabilities that are greater than* 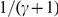 *(Theorem 3), and for* 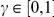 *it can be given as the consensus estimator calculated from* *Eq. (8)* *(Corollary 1). For* 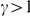 *, the* 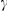 *-centroid estimator is obtained with the aid of a dynamic programming algorithm with the same type of iterations as in the Nussinov algorithm: *

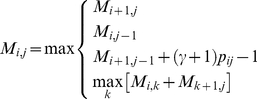
(10)
*where* 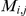 *stores the best score of the sub-sequence* 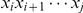 *(see “Pairwise alignment of biological sequences (Problem 1)” in* *Appendices* *for the detail descriptions).*

The 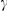-centroid estimators are implemented in LAST [4] for Problem 1 and in CentroidFold [12], [15] for Problem 2.

**Problem 4 (Estimation of phylogenetic trees)** *Given a set of operational taxonomic units* 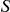 *, predict their phylogenetic trees (unrooted and multi-branched trees) as a point in* 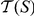 *, the space of all the possible phylogenetic trees of* 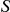 *.*

The phylogenetic tree in 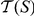 is represented as a binary vector with 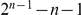 dimension where 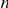 is the number of units in 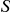, based on partition of 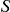 by cutting every edge in the tree (see “The space of phylogenetic trees: 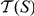
” in Appendices for details). A sampling algorithm can be used to estimate the partitioning probabilities approximately [16].

**Example 5 (** 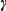 **-centroid estimator of phylogenetic estimation)** *In Problem 4 with Assumption 1, the* 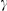 *-centroid estimator maximizes the number of the partitioning probabilities which are greater than* 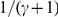 *(Theorem 3), and for* 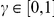 *it can be give as the consensus estimator calculated from* *Eq. (8)* *(Corollary 1) (see “Estimation of phylogenetic trees (Problem 4)” in* *Appendices* *for details).*

Because the Hamming distance between two trees in 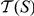 is known as topological distance [17], the 1-centroid estimator minimizes the expected topological distance. In contrast to Example 3 and Example 4, it appears that no method can efficiently compute the 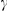-centroid estimator with 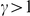 in Example 5. Despite the difficulties of the application to phylogenetic trees, recently, a method applying the concept of generalized centroid estimators was developed [18].

### Generalized centroid estimators for representative prediction

Predictions based on probability distributions on the predictive space were discussed in the previous sections. However, there are certain even more complex problems in bioinformatics, as illustrated by the following example.

**Problem 5 (Prediction of common secondary structures of RNA sequences)** *Given a set of RNA sequences* 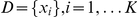 *and their multiple alignment of length* 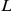 *and the same energy model for each RNA sequence, predict their common secondary structure as a point in* 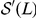 *, which is the space of all possible secondary structures of length* 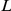 *.*

In the case of Problem 5, although the probability distribution is not implemented in the predictive space, each RNA sequence 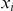 has a probability distribution on its secondary structure derived from the energy model. Therefore, the theories presented in the previous section cannot be applied directly to this problem. However, if we devise a new type of gain function that connects the predictive space with the parameter space of the secondary structure of each RNA sequence, we can calculate the expected gain over the distribution on the parameter spaces of RNA sequences. In order to account for this type of problem in general, we introduce Assumption 2 and Definition 8 as follows.

**Assumption 2** *In Problem 3 there exists a probability distribution* 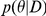 *on the parameter space* 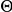 *which might be different from the predictive space* 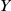 *.*

**Definition 8 (Generalized gain function)** *In Problem 3 with Assumption 2, for a point* 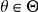 *and a prediction* 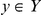 *, a generalized gain function is defined as* 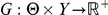 *,* 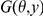 *.*

It should be emphasized that the MEG estimator (Definition 3), pointwise gain function (Definition 4) and Theorem 1 can be extended to the generalized gain function.

In the case of Problem 5, for example, the parameter space is the product of the spaces of the secondary structures of each RNA sequence, and the probability distribution is the product of the distributions of secondary structures of each RNA sequence. Here, the general form of the problem of representative prediction is introduced.

**Problem 6 (Representative prediction)** *In Problem 3 with Assumption 2, if the parameter space is represented as a product space (* 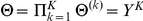 *) and the distribution of* 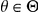 *has the form* 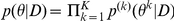 *, predict a point* 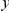 *in the predictive space* 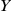 *.*

The generalized gain function for the representative prediction should be chosen such that the prediction reflects as much as each data entry. Therefore, it is natural to use the following generalized gain function that integrates the gain for each parameter.

**Definition 9 (Homogeneous generalized gain function)** *In Problem 6, a homogeneous generalized gain function is defined as*

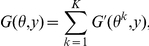

*where* 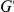 *is the gain function in Definition 2.*

**Definition 10 (Representative estimator)** *In Problem 6, given a homogeneous generalized gain function* 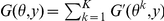 *, the MEG estimator defined as *

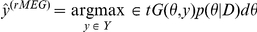

*is referred to as the representative estimator.*

**Proposition 1** *The representative estimator is equivalent to an MEG estimator with averaged probability distribution on the predictive space* 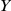 *:*

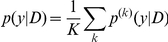

*and a gain function* 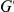 *.*

This proposition shows that a representative prediction problem with any homogeneous generalized gain function can be solved in a manner similar to Problem 3 (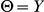) with averaged probability distribution. Therefore, the 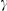-centroid estimator for a representative prediction satisfies Corollary 2.

**Corollary 2** *In Problem 6, the representative estimator where* 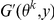 *is the gain function of the* 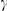 *-centroid estimator on* 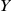 *, is the* 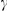 *-centroid estimator for the averaged probability distribution and satisfies the same properties in Theorem 2, Theorem 3, and Corollary 1.*

### Estimators based on marginal probabilities

In the previous section, we introduced Assumption 2, where there is a parameter space 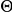 that can be different from the predictive space 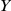, and we discussed the problem of representative prediction. In this section, we discuss another type of problems where 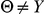. An example is presented below.

**Problem 7 (Pairwise alignment using homologous sequences)** *Given a data set* 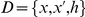 *, where* 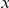 *and* 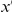 *are two biological sequences to be aligned and* 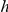 *is a sequence that is homologous to both* 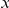 *and* 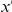 *, predict a point* 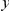 *in the predictive space* 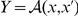 *(the space of all possible alignments of* 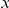 *and* 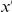 *).*

The precise probabilistic model of this problem might include the phylogenetic tree, ancestor sequences and their alignments. Here, we assume a simpler situation where the probability distribution of all possible multiple alignments of 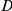 is given. We predict the pairwise alignment of two specific sequences according to the probability distribution of multiple alignments. Although the parameter space 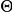, which is the space of all the possible multiple alignments, can be parametrized using the parameters of the spaces of the alignments of all pairs that can be formed from the sequences in 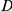, 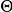 itself is not the product space of these spaces because these pairwise alignments are not independent: for 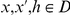, 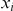 must be aligned to 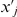 if both 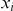 and 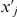 are aligned to 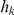. This type of problems can be generalized as follows.

**Problem 8 (Prediction in a subspace of the parameter space)** *In Problem 3 with Assumption 2, if the parameter space* 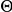 *is represented as* 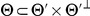 *, predict a point* 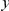 *in the predictive space* 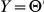 *.*

For the problem of representative prediction (Problem 6), generalized gain functions on 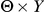 were introduced (Definition 8 and Definition 9). In contrast, in Problem 8, the values of the parameters in 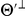 are not important, and a point in 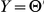 is predicted. In Problem 7, for example, the optimal multiple alignment of 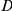, the pairwise alignment of 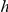 and 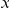, and the pairwise alignment of 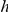 and 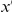 are irrelevant, but instead we predict the pairwise alignment of 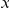 and 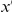. The MEG estimator for the gain function defined on 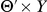 can be written as 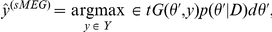
where 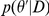 on 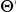 is the marginalized distribution 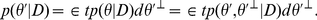
(11)


From the above MEG estimator, it might appear that Problem 8 is trivial. However, it is not a simple task to calculate the marginalized distribution in Eq. (11) in actual problems.

To reduce the computational cost, we change Problem 8 by introducing an approximated probability distribution on the product space 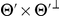 a follows.

**Problem 9 (Prediction in product space)** *In Problem 3 with Assumption 2, if the parameter space* 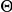 *is represented as* 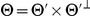 *and the probability distribution on* 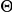 *is defined as *

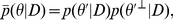
(12)
*predict a point* 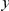 *in the predictive space* 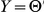 *.* **

This factorization of spaces and probability distributions creates a number of inconsistencies in the parameter space with respect to the original Problem 8. In other words, the approximated distribution yields non-zero values for a point that is not included in the original 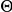 (in Problem 8) but in 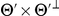. To reduce these inconsistencies, a new type of gain function and a new estimator are introduced as follows.

**Definition 11 (** 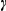 **-type pointwise gain function)** *In Problem 8, a* 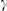 *-type pointwise gain function is defined as* 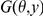 *in* *Eq. (1)* *in Definition 4 having*

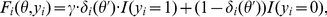
(13)
*where the value* 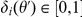 *in the gain function should be designed to reduce the inconsistencies resulting from the factorization*.

**Definition 12 (Approximated** 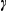 **-type estimator)** *In Problem 9, with a* 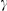 *-type pointwise gain function with* 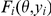 *in* *Eq. (13)* *on* 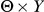 *, an approximated* 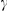 *-type estimator is defined as an MEG estimator:*

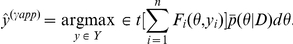



**Example 6 (PCT in pairwise alignment)** *We obtain the approximate estimator for Problem 7 with the following settings. The parameter space is given as* 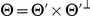 *, where *

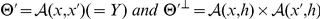

*and the probability distribution on the parameter space* 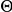 *is given as *

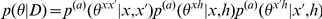

*for* 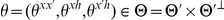 *. The* 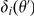 *in* *Eq. (13)* *of the* 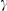 *-type pointwise gain function is defined as*

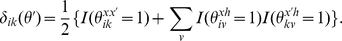



*The approximated* 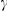 *-type estimator for this* 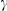 *-type pointwise gain function is employed in a part of* probabilistic consistency transformation *(PCT)* [*19*] *, which is an important step toward accurate multiple alignments. See “Pairwise alignment using homologous sequences” in* *Appendices* *for precise descriptions.*

It is easily seen that Theorem 3 applies to the approximated 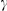-type estimator if 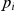 in Theorem 3 is changed as follows: 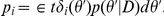



Moreover, to confirm whether approximated 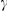-type estimator contains the consensus estimator for the same gain function, it is only necessary to check if 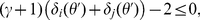
(14)instead of Eq. (3) in Theorem 1. (Note that Theorem 1 can be extended to the *generalized* (pointwise) gain function: see Theorem 4.)

## Discussion

### Properties of the 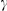-centroid estimator

In this paper, general criteria for designing estimators are given by the maximum expected gain (MEG) estimator (Definition 3). The Bayesian ML estimator is an MEG estimator with the delta function 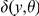 as the gain function, which means that only the probability for the “perfect match” is counted. To overcome the drawbacks of the Bayesian ML estimator, the centroid estimator [1] takes into account the overall ensemble of solutions and minimizes the expected Hamming loss. Because the Hamming loss is not the standard evaluation measures for actual problems, we have proposed an estimator of a more general type, the 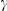-centroid estimator (Definition 7), which includes the centroid estimator as a special case, 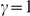. The 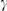-centroid estimator is an MEG estimator that maximizes the expected value of 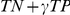, which generally covers all possible linear combination of the numbers of true positives (TP), true negatives (TN), false positives (FP) and false negatives (FN) (Theorem 2). Since most of the evaluation measures of the prediction accuracy are functions of these numbers [20], the 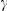-centroid estimator is related to the principle of maximum expected accuracy (MEA). It should be noted that MEG estimators have been proposed that are similar to the 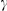-centroid estimator for some specific problems, for example, the alignment metric accuracy (AMA) estimator [21] (see Appendices for the formal definition) for pairwise alignment (Problem 1) and the MEA-based estimator [5] (see Appendices for the formal definition) for prediction of secondary structure of RNA (Problem 2). However, these estimators display a *bias* with respect to the accuracy measures for the problem (see Eqs. (20) and (22)), and are therefore inappropriate from the viewpoint of the principles of MEA. Moreover, these estimators cannot be introduced in a general setting, that is, Problem 3. It has been also shown that the 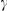-centroid estimator outperforms the MEA-based estimator [5] for various probability distributions in computational experiments [12]. (See “Pairwise alignment of biological sequences (Problem 1)” and “Secondary structure prediction of an RNA sequence (Problem 2)” in Appendices for relations between the 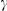-centroid estimator and other estimators in Problems 1 and 2, respectively.)

### How to determine the parameter in 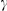-centroid estimator

The parameter 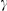 in 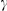-centroid estimators adjusts sensitivity and PPV (whose relation is tradeoff). MCC or F-score is often used to obtain a balanced measure between sensitivity and PPV. In RNA secondary structure predictions, it has been confirmed that the best 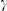 (with respect to MCC) of the 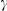-centroid estimator with CONTRAfold model was larger than that with McCaskill model [12]. It shows that the best 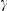 (with respect to a given accuracy measure) depends on not only estimation problems but also probabilistic models for predictive space. The parameter 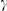 trained by using reference structures was therefore employed as the default parameter in CentroidFold [12]. In order to select the parameter automatically (with respect to a given accuracy measure such as MCC and F-score), an approximation of maximizing expected MCC (or F-score) with the 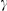-centroid estimator can be utilized [22].

### Accuracy measures and computational efficiency

The reader might consider that it is possible to design estimators that maximize the expected MCC or F-score which balances sensitivity (SEN) and positive predictive value (PPV). However, it is much more difficult to compute such estimators in comparison with the 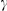-centroid estimator, as described below.

The expected value of the gain function of the 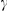-centroid estimator can be written with marginalized probabilities as in Eq. (7), which can be efficiently computed by dynamic programming in many problems in bioinformatics, for example, the forward-backward algorithm for alignment probabilities and the McCaskill algorithm for base pairing probabilities. Under a certain condition of the predictive space, which many problems in bioinformatics satisfy, the 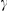-centroid estimator maximizes the sum of marginalized probabilities greater than 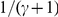 (Theorem 3). Moreover, under an additional condition of the predictive space and the pointwise gain function, which again many problems in bioinformatics satisfy, the 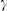-centroid estimators for 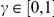 can be easily calculated as the consensus estimators, which collect in the binary predictive space the components that have marginalized probabilities greater than 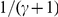 (Corollary 1). For 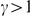, there often exist dynamic programming algorithms that can efficiently compute the 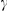-centroid estimators (Examples 4 & 3), but there are certain problems, such as Problem 4, which seem to have no efficient dynamic programming algorithms.

The gain function of the estimators that maximize MCC or F-score, and also SEN or PPV contain *multiplication* and/or *division* of TP, TN, FP and FN, while the gain function of the 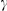-centroid estimator contains only the weighted *sums* of these values (i.e., 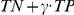). Therefore, the expected gain is not written with marginalized probabilities as in Eq. (7), and it is difficult to design efficient computational algorithms for those estimators. In predicting secondary structures of RNA sequences (Problem 2), for example, it is necessary to enumerate all candidate secondary structures or sample secondary structures for an approximation in order to compute the expected MCC/F-score of a predicted secondary structure.

### Probability distributions are not always defined on predictive space

After discussing the standard estimation problems on a binary space where the probability distribution is defined on the predictive space, we have proposed a new category of estimation problems where the probability distribution is defined on a parameter space that differs from the predictive space (see Assumption 2). Two types of estimators for such problems, for example, estimators for representative prediction and estimators based on marginalized distribution, have been discussed.

Prediction of the common secondary structure from an alignment of RNA sequences (Problem 5) is an example of representative prediction. The probability distribution is not implemented in the predictive space, the space of common secondary structure, but each RNA sequence has a probability distribution for its secondary structure. Because the “correct” reference for the common secondary structure is not known in general, direct evaluation of the estimated common secondary structure is difficult. In the popular evaluation process for this problem, the predicted common secondary structure is mapped to each RNA sequence and compared to its reference structure. Using the homogeneous generalized gain function exactly implements this evaluation process and the MEG estimator for the averaged probability distribution is equivalent to the MEG estimator for homogeneous generalized gain function. Therefore, we can use the averaged base pairing probabilities according to the alignment as the distribution for the common secondary structure (see “Common secondary structure prediction from a multiple alignment of RNA sequences” in Appendices for detailed discussion). The representative estimator for Problem 5 is implemented in software CentroidAlifold. Another example of representative prediction is the “alignment of alignments” problem, which is the fundamental element of progressive multiple alignment of biological sequences. The evaluation process using the sum of pairs score corresponds to using the homogeneous generalized gain function. (see “Alignment between two *alignments* of biological sequences” in Appendices for detailed discussion).

Estimation problems of marginalized distributions can be formalized as prediction in a subspace of the parameter space (Problem 8). If we can calculate the marginalized distribution on the predictive space from the distribution on the parameter space, all general theories apply to the predictive space and the marginalized distribution. In actual problems, such as pairwise alignment using homologous sequences (Problem 7), however, computational cost for calculation of the marginalized probability is quite high. We introduced the factorized probability distribution (Eq. (12)) for approximation, the 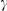-type pointwise gain function (Definition 11) to reduce the inconsistency caused by the factorization, and the approximated 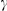-type estimator (Definition 12). In Problem 7, the probability consistency transformation (PCT), which is widely used for multiple sequence alignment, is interpreted as an approximated 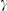-type estimator. Prediction of secondary structures of RNA sequences on the basis of homologous sequences [23] (see Problem 13 in Appendices) and pairwise alignment for *structured* RNA sequences are further examples of this type of problems.

### Application of 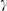-centroid estimator to cluster centroid

In case probability distribution on the predictive space is multi-modal, 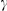-centroid estimators can provide unreliable solutions. For example, when there are two clusters of secondary structures in predictive spaces and those structures are exclusive, the 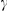-centroid estimator might give a “chimeric” secondary structure whose free energy is quite high. To avoid this situation, Ding *et al.* [24] proposed a notion of the *cluster centroid*, which is computed by the centroid estimator with a given cluster in a predictive space. We emphasize that the extension of cluster centroid by using 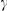-centroid estimator is straightforward and would be useful.

### Conclusion

In this work, we constructed a general framework for designing estimators for estimation problems in high-dimensional discrete (binary) spaces. The theory is regarded as a generalization of the pioneering work conducted by Carvalho and Lawrence, and is closely related to the concept of MEA. Furthermore, we presented several applications of the proposed estimators (see Table 1 for summary) and the underlying theory. The concept presented in this paper is highly extendable and sheds new light on many problems in bioinformatics. In future research, we plan to investigate further applications of the 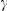-centroid and related estimators presented in this paper.

**Table 1:** Summary of applications in bioinformatics.

Alignment	(1) Pairwise alignment of biological sequences	(4) Pairwise alignment of two multiple alignments	(6) Pairwise alignment using homologous sequences	
Section	Section	Section	Section	
Data 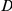	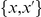	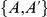	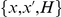	
Predictive space 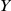	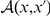	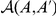	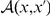	
Parameter space 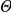	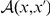	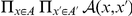	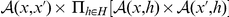	
Probability 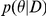	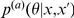	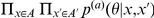	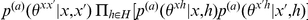	
Type of estimator	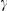 -centroid	representative	approximate	
Software	LAST	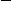	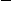	
Reference	[4]	[19], This work	[19], This work	
RNA	(2) Secondary structure prediction of RNA	(5) Common secondary structure prediction	(7) Secondary structure prediction using homologous sequences	(8) Pairwise alignment of structured RNAs
Section	Section	Section	Section	Section
Data 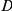	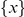	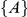	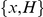	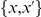
Predictive space 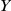	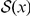	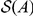	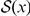	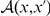
Parameter space 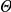	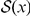	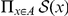	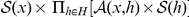	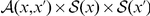
Probability 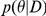	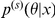	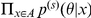	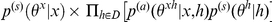	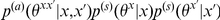
Type of estimator	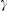 -centroid	representative	approximate	approximate
Software	CentroidFold	CentroidAlifold	CentroidHomfold	CentroidAlign
Reference	[12]	[12], [49]	[23]	[52]
Phylogenetic tree	(3) Estimation of phylogenetic tree			
Section	Section			
Data 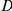	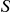			
Parameter space 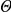	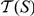			
Predictive space 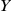	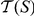			
Probability 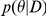	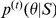			
Type of estimator	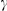 -centroid			
Reference	This work			

The top row includes problems about RNA secondary structure predictions and the middle row includes problems about alignment of biological sequences. Note that the estimators in the same column corresponds to each other.

## Appendices

### Discrete (binary) spaces in bioinformatics

In this section, we summarize three discrete spaces that appear in this paper. These discrete spaces are often used in the definition of the predictive spaces and the parameter spaces. It should be noted that every discrete space described below is identical in form to Eq. (2).

#### The space of alignments of two biological sequences: 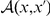


We define a space of the alignments of two biological (DNA, RNA and protein) sequences 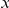 and 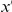, denoted by 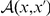, as follows. We set 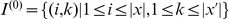 as a base index set, and a binary variable 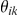 for 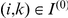 is defined by 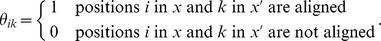



Then 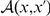 is a subset of 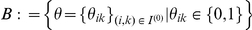 and is defined by
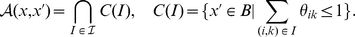



Here 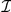 is a set of index-sets:



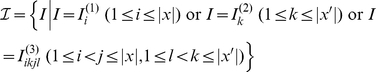
where
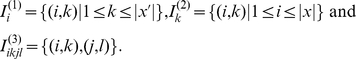



The inclusion 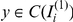 means that position 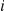 in the sequence 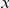 aligns with *at most one* position in the sequence 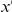 in the alignment 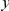, 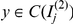 means that position 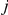 in the sequence 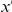 aligns with *at most one* position in the sequence 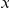 and 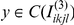 means the alignment 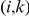 and 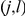 is *not crossing*. Note that 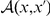 depends on only the length of two sequences, namely, 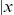 and 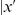.

#### The space of secondary structures of RNA: 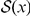


We define a space of the secondary structures of an RNA sequence 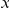, denoted by 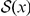, as follows. We set 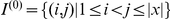 as a base index set, and a binary variable 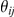 for 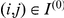 is defined by 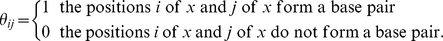



Then 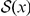 is a subset of 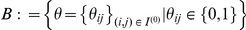 and is defined by 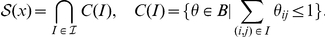



Here 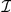 is a set of index-sets 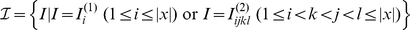
where
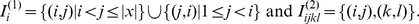



The inclusion 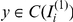 means that position 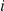 in the sequence 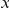 belongs to *at most one* base-pair in a secondary structure 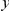, and 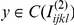 means two base-pairs whose relation is *pseudo-knot* are not allowed in 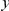. Note that 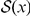 depends on only the length of the RNA sequence 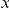, that is, 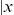.

#### The space of phylogenetic trees: 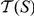


We define a space of phylogenetic trees (unrooted and multi-branch trees) of a set of 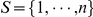, denoted by 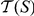, as follows. We set 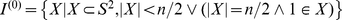, where 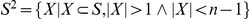, as a base index set and we define binary variables 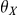 for 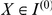 by 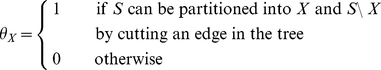



Then 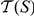 is a subset of 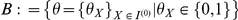 and is defined by
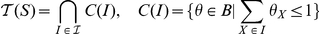
where 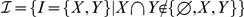. Note that 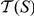 depends on only the number of elements in 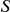. We now give several properties of 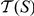 that follow directly from the definition.

**Lemma 1** *The number of elements in* 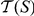 *(i.e.* 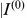 *) is equal to* 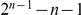 *where* 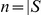 *.*

**Lemma 2** *The* topological distance [*17*] *between two phylogenetic trees* 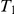 *and* 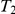 *in* 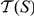 *is*

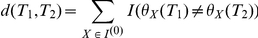

*where* 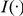 *is the indicator function.*

**Remark 1** *If we assume the additional condition* 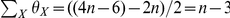 *, then* 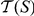 *is a set of binary trees.*

### Probability distributions on discrete spaces

We use three probability distributions in this paper.

#### Probability distributions 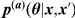 on 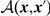


For two protein sequences 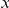 and 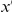, a probability distribution 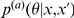 over the space 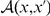, which is the space of pairwise alignments of 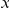 and 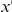 defined in the previous section, is given by the following models.

1. Miyazawa model [13] and Probalign model [25]: 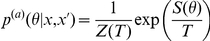
where 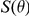 is the score of an alignment 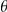 under the given scoring matrix (We define 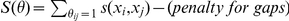 where 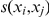 is a score for the correspondence of bases 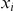 and 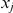), 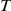 is the thermodynamic temperature and 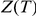 is the normalization constant, which is known as a *partition function*.
2. Pair Hidden Markov Model (pair HMM) [19]: 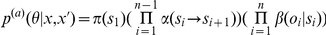
where 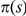 is the initial probability of starting in state 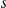, 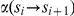 is the transition probability from 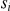 to 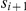 and 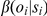 is the omission probability for either a single letter or aligner residue pair 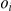 in the state 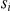.
3. CONTRAlign (pair CRF) model [26]: 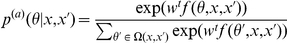
where 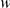 is a parameter vector and 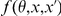 is a vector of features that indicates the number of times each parameter appears, 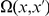 denotes the set of all possible alignments of 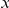 and 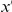. We do not describe the feature vectors and refer readers to the original paper [26].

**Remark 2** *Strictly speaking, the alignment space in the pair hidden Markov model and the CONTRAlign model consider the patterns of gaps. In these cases, we obtain the probability space on* 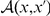 *by a marginalization.*

#### Probability distributions 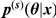 on 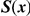


For an RNA sequence 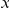, a probability distribution 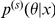 over 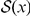, which is the space of secondary structures of 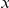 defined in the previous section is given by the following models.

1. McCaskill model [14]: This model is based on the energy models for secondary structures of RNA sequences and is defined by 
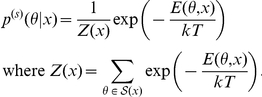
 where 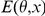 denotes the energy of the secondary structure that is computed using the energy parameters of Turner Lab [27], 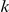 and 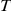 are constants and 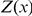 is the normalization term known as the *partition function*.
2. Stochastic Context free grammars (SCFGs) model [28]: 
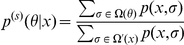
where 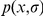 is the joint probability of generating the parse 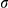 and is given by the product of the transition and emission probabilities of the SCFG model and 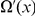 is all parses of 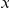, 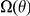 is all parses for a given 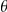.
3. CONTRAfold (CRFs; conditional random fields) model [5]: This model gives us the best performance on secondary structure prediction although it is not based on the energy model. 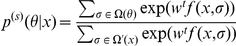
where 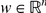, 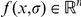 is the feature vector for 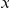 in parse 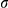, 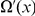 is all parses of 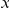, 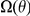 is all parses for a given 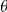.

#### Probability distributions 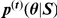 on 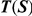


A probability distribution 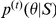 on 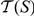 is given by probabilistic models of phylogenetic trees, for example, [29], [ 30]. Those models give a probability distribution on binary trees and we should marginalize these distributions for multi-branch trees.

### Evaluation measures defined using TP, TN, FP and FN

There are several evaluation measures of a prediction in estimation problems for which we have a reference (correct) prediction in Problem 3. The Sensitivity (SEN), Positive Predictive Value (PPV), Matthew's correlation coefficient (MCC) and F-score for a prediction are defined as follows. 
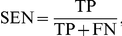


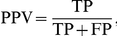


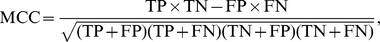


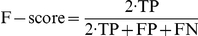
where TP (the number of true positive), TN (the number of true negative), FP (the number of false positive) and FN (the number of false negative) are defined by 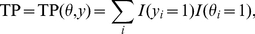
(15)

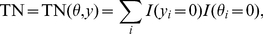
(16)

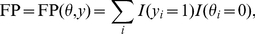
(17)

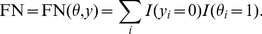
(18)


It should be noted that these measures can be written as a function of TP, TN, FP and FN. See [20] for other evaluation measures.

### Schematic diagrams of representative and approximated 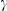-type estimators

**Figure 1:**
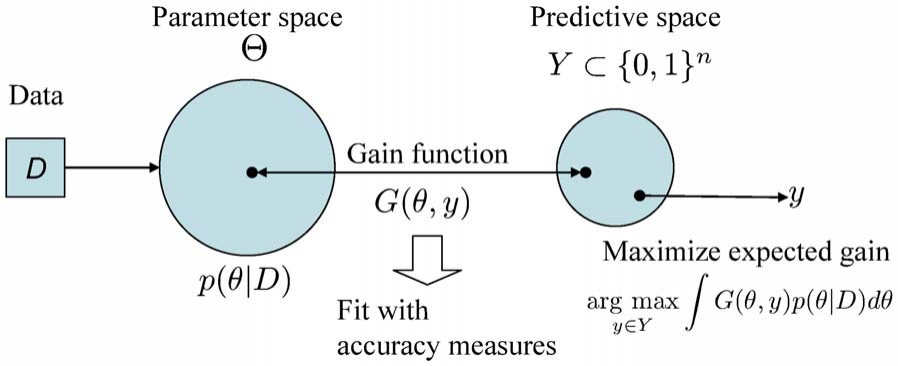
Schematic diagram of the MEG estimator (Definition 3).

**Figure 2:**
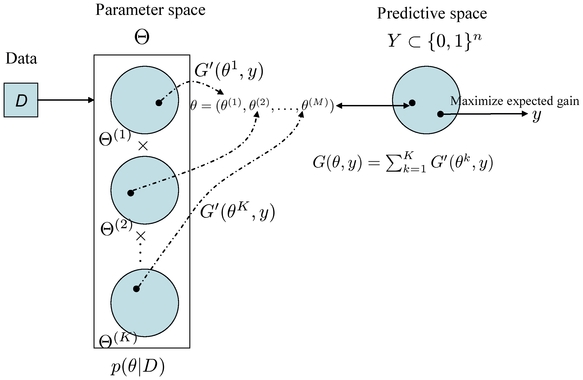
Schematic diagram of the representative estimator (Definition 10). The parameter space 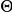 is a product space and is different from the predictive space 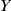.

**Figure 3:**
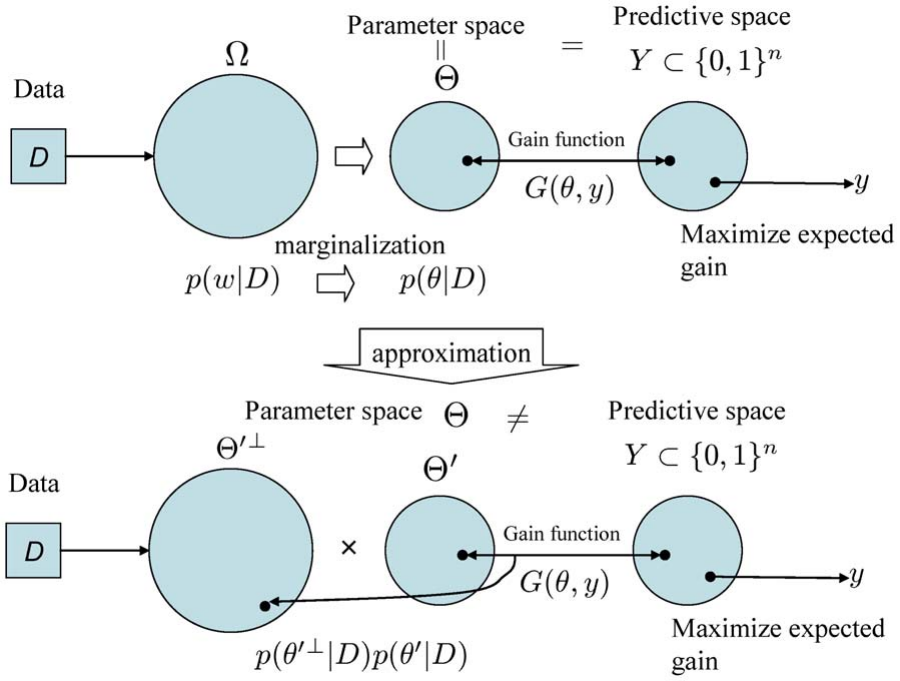
Schematic diagram of the approximated 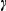-type estimator (Definition 12). The estimator in the top figure shows the 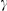-centroid estimator with the marginalized probability distribution, and the one in the bottom figure shows its approximation.

The schematic diagrams of the MEG estimator (Definition 3), the representative estimator (Definition 10) and the approximated 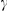-type estimator (Definition 12) are shown in Figure 1, Figure 2 and Figure 3, respectively.

### Applications in bioinformatics

In this section we describe several applications to bioinformatics of the general theories. Some of these applications have already been published. In those cases, we briefly explain the applications and the readers should see the original paper for further descriptions as well as the computational experiments. All of the applications in this section are summarized in Table 1.

#### Pairwise alignment of biological sequences (Problem 1)

The pairwise alignment of biological (DNA, RNA, protein) sequences (Problem 1) is another fundamental and important problem of sequence analysis in bioinformatics (cf. [31]).

The 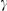-centroid estimator for Problem 1 can be introduced as follows:

**Estimator 1 (** 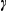 **-centroid estimator for Problem∼:align)** *For Problem 1, we obtain the* 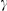 *-centroid estimator where the predictive space* 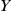 *is equal to* 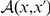 *and the probability distribution on* 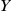 *is taken by* 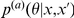 *.*

First, Theorem 2 and the definition of 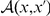 lead to the following property.

**Property 1 (A relation of Estimator 1 with accuracy measures)** *The* 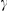 *-centroid estimator for Problem 1 is suitable for the accuracy measures: SEN, PPV, MCC and F-score* with respect to the aligned-bases *in the predicted alignment.*

Note that accurate prediction of aligned-bases is important for the analysis of alignments, for example, in phylogenetic analysis. Therefore, the measures in above are often used in evaluations of alignments e.g. [4].

The marginalized probability 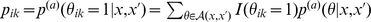 is called the *aligned-base (matching) probability* in this paper. The aligned-base probability matrix 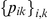 can be computed by the forward-backward algorithm whose time complexity is equal to 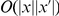 [31]. Now, Theorem 3 leads to the following property.

**Property 2 (Computation of Estimator 1)** *The pairwise alignment of Estimator 1 is found by maximizing the sum of aligned-base probabilities* 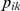 *(of the aligned-bases in the predicted alignment) that are larger than* 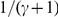 *. Therefore, it can be computed by a Needleman-Wunsch-style dynamic programming (DP) algorithm* [*32*] *after calculating the aligned-base matrix* 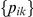 *:*

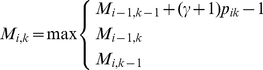
(19)
*where* 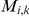 *stores the optimal value of the alignment between two sub-sequences,* 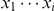 *and* 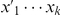 *.*

The time complexity of the recursion of the DP algorithm in Eq. (19) is equal to 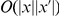, so the total computational cost for predicting the secondary structure of the 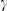-centroid estimator remains 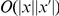.

By using Corollary 1, we can predict the pairwise alignment of Estimator 1 with 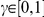 without using the DP algorithm in Eq. (19).

**Property 3 (Computation of Estimator 1 with** 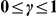 **)** *The pairwise alignment of the* 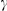 *-centroid estimator can be predicted by collecting the aligned-bases whose probabilities are larger than* 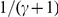 *.*

The genome alignment software called LAST (http://last.cbrc.jp/) [4], [ 33] employs the 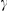-centroid estimator accelerated by an X-drop algorithm, and the authors indicated that Estimator 1 reduced the false-positive aligned-bases, compared to the conventional alignment (maximum score estimator).

Relations of Estimator 1 with existing estimators are summarized as follows:

1. A relation with the estimator by Miyazawa [13] (i.e. the centroid estimator): Estimator 1 where 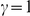 and the Miyazawa model is equivalent to the centroid estimator proposed by Miyazawa [13].
2. A relation with the estimator by Holmes *et al.* [34]: Estimator 1 with sufficiently large 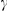 is equivalent to the estimator proposed by Holmes *et al.*, which maximizes the sum of matching probabilities in the predicted alignment.
3. A relation with the estimator in ProbCons: In the program, ProbCons, Estimator 1 with pair HMM model and the sufficient large 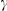 was used. This means that ProbCons only take care the sensitivity (or SPS) for the predicted alignment.
4. A relation with the estimator by Schwartz *et al.*: For Problem 1, Schwartz *et al.* [21] proposed an Alignment Metric Accuracy (AMA) estimator, which is similar to the 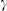-centroid estimator (see also [3]). The AMA estimator is a maximum gain estimator (Definition 3) with the following gain function. 
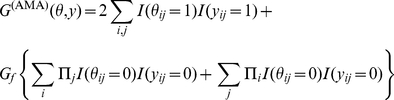
for 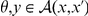. In the above equation, 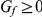 is a gap factor, which is a weight for the prediction of gaps. We refer to the function 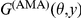 as the gain function of the AMA estimator. In a similar way to that described in the previous section, we obtain a relation between 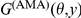 and 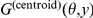 (the gain function of the 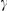-centroid estimator). If we set 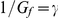, then we obtain 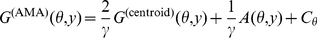
(20)where
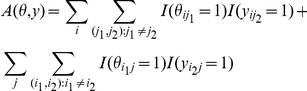
and 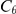 is a value which does not depend on 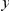. If 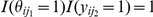 for 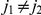, then we obtain 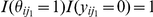 and 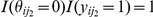, and this means that 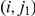 is an aligned pair that is a false negative and 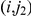 is an aligned pair that is a false positive when 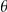 is a reference alignment and 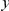 is a predicted alignment. Therefore, the terms 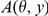 (in Eq. (20)) in the gain function of AMA are not appropriate for the evaluation measures SEN, PPV, MCC and F-score for aligned bases. In summary, the 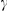-centroid estimator is suitable for the evaluation measures: SEN, PPV and F-score with respect to the aligned-bases while the AMA estimator is suitable for the AMA.

#### Secondary structure prediction of an RNA sequence (Problem 2)

Secondary structure prediction of an RNA sequence (Problem 2) is one of the most important problems of sequence analysis in bioinformatics. Its importance has increased due to the recent discovery of functional non-coding RNAs (ncRNAs) because the functions of ncRNAs are closely related to their secondary structures [35].


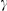
-centroid estimator for Problem 2 can be introduced as follows:

**Estimator 2 (** 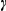 **-centroid estimator for Problem 2)** *For Problem 2, we obtain the* 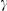 *-centroid estimator (Definition 7) where the predictive space* 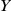 *is equal to* 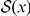 *and the probability distribution on* 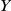 *is taken by* 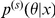 *.*

The general theory of the 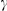-centroid estimator leads to several properties. First, the following property is derived from Theorem 2 and the definition of 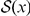.

**Property 4 (A relation of Estimator 2 with accuracy measures)** *The* 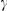 *-centroid estimator for Problem 2 is suitable for the widely-used accuracy measures of the RNA secondary structure prediction: SEN, PPV and MCC with respect to base-pairs in the predicted secondary structure.*

Because the base-pairs in a secondary structure are biologically important, SEN, PPV and MCC with respect to base-pairs are widely used in evaluations of RNA secondary structure prediction, for example, [5], [ 12], [ 36].

The marginalized probability 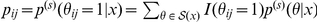 is called a *base-pairing probability*. The base-paring probability matrix 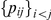 can be computed by the Inside-Outside algorithm whose time complexity is equal to 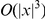 where 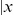 is the length of RNA sequence 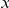 [14], [ 31]. Then, Theorem 3 leads to the following property.

**Property 5 (Computation of Estimator 2)** *The secondary structure of Estimator 2 is found by maximizing the sum of the base-pairing probabilities* 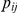 *(of the base-pairs in the predicted structure) that are larger than* 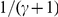 *. Therefore, it can be computed by a Nussinov-style dynamic programming (DP) algorithm* [*37*] *after calculating the base-pairing probability matrix* 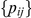 *:*

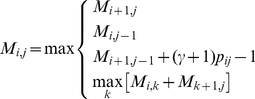
(21)
*where* 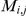 *stores the best score of the sub-sequence* 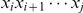 *.*

If we replace “
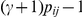
” with “
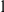
” in Eq. (21), the DP algorithm is equivalent to the Nussinov algorithm [37] that maximizes the number of base-pairs in a predicted secondary structure. The time complexity of the recursion of the DP algorithm in Eq. (21) is equal to 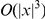. Hence, the total computational cost for predicting the secondary structure of the 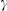-centroid estimator remains 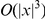, which is the same time complexity as for standard software: Mfold [38], RNAfold [39] and RNAstructure [40].

By using Corollary 1, we can predict the secondary structure of Estimator 2 with 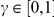 without using the DP algorithm in Eq. (21).

**Property 6 (Computation of Estimator 2 with** 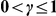 **)** *The secondary structure of the* 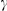 *-centroid estimator with* 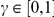 *can be predicted by collecting the base-pairs whose probabilities are larger than* 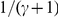 *.*

The software CentroidFold [12], [ 15] implements Estimator 2 with various probability distributions for the secondary structures, such as the CONTRAfold and McCaskill models.

Relations of Estimator 2 with other estimators are summarized as follows:

1. A relation with the estimator used in Sfold [41], [ 42]: Estimator 2 with 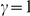 and the McCaskill model (i.e. the centroid estimator with the McCaskill model) is equivalent to the estimator used in the Sfold program.
2. A relation with the estimator used in CONTRAfold: For Problem 2, Do *et al.* [5] proposed an MEA-based estimator, which is similar to the 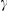-centroid estimator. (The MEA-based estimator was also used in a recent paper [6].) The MEA-based estimator is defined by the maximum expected gain estimator (Definition 3) with the following gain function for 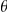 and 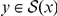. 
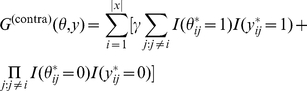
(22)where 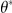 and 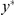 are symmetric extensions of (upper triangular matrices) 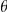 and 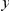, respectively (i.e. 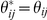 for 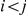 and 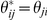 for 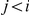; the definition of 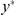 is similar.). It should be noted that, under the general estimation problem of Problem 3, the gain function of Eq. (22) cannot be introduced, and the gain function is specialized for the problem of RNA secondary structure prediction. The relation between the gain function of the 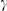-centroid estimator (denoted by 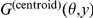 and defined in Definition 7) and the one of the MEA-based estimator is 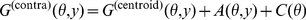
(23)where the additional term 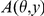 is positive for *false* predictions of base-pairs (i.e., 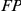 and 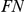) and 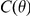 does not depend on the prediction 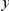 (see [12] for the proof). This means the MEA-based estimator by Do et al. possess a bias against the widely-used accuracy measures for Problem 2 (SEN, PPV and MCC of base-pairs) compared with the 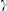-centroid estimator. Thus, the 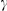-centroid estimator is theoretically superior to the MEA-based estimator by Do et al. with respect to those accuracy measures. In computational experiments, the authors confirmed that the 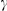-centroid estimator is always better than the MEA-based estimator when we used the same probability distribution of secondary structures. See [12] for details of the computational experiments.

#### Estimation of phylogenetic trees (Problem 4)

The 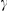-centroid estimator for Problem 4 can be introduced as follows:

**Estimator 3 (** 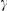 **-centroid estimator for Problem 4)** *For Problem 4, we obtain the* 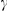 *-centroid estimator (Definition 7) where the predictive space* 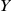 *is equal to* 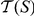 *and the probability distribution on* 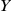 *is taken by* 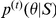 *.*

The following property is easily obtained by Theorem 2 and [17].

**Property 7 (Relation of 1-centroid estimator and topological distance)** *The* 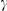 *-centroid estimator with* 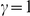 *(i.e. centroid estimator) for Problem 4 minimizes expected topological distances.*

For 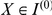 (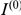 is a set of partitions of 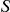 and is formally defined in the previous section), we call the marginalized probability 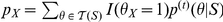 *partitioning probability*. However, it is difficult to compute 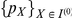 as efficiently as in the prediction of secondary structures of RNA sequences, where it seems possible to compute the base-pairing probability matrix in polynomial time by using dynamic programming). Instead, a sampling algorithm can be used for estimating 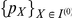 approximately [16] for this problem. Once 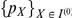 is estimated, Theorem 3 leads to the following:

**Property 8 (Computaion of Estimator 3)** *The phylogenetic tree of Estimator 3 is found by maximizing the sum of the partitioning probabilities* 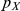 *(of the partitions given by the predicted tree) that are larger than* 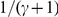 *.*

In contrast to Estimator 1 (the 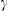-centroid estimator for secondary structure prediction of RNA sequence) and Estimator 2 (the 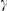-centroid estimator for pairwise alignment), it appears that there is no efficient method (such as dynamic programming algorithms) to computed Estimator 3 with 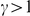. Estimator 1 with 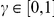, however, can be computed by using the following property, which is directly proven by Corollary 1 and the definition of the space 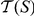.

**Property 9 (Estimator 3 with** 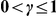 **)** *The* 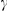 *-centroid estimator with* 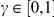 *for Problem 4 contains its consensus estimator.*

#### Alignment between two *alignments* of biological sequences

**Figure 4:**
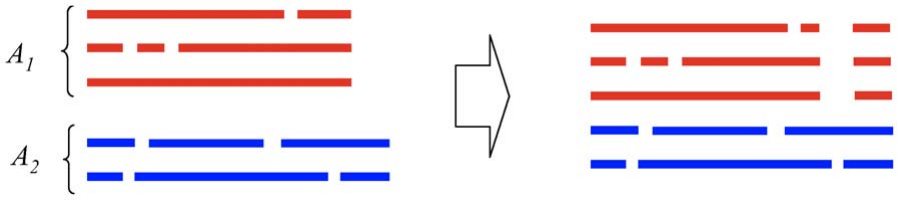
Alignment between two multiple alignments 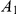 and 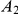 (Problem 10).

In this section we consider the problem of the alignment between two multiple alignments of biological sequences (Figure 4), which is often important in the multiple alignment of RNA sequences [19]. This problem is formulated as follows.

**Problem 10 (Alignment between two alignments of biological sequences)** *The data is represented as* 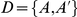 *where* 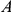 *and* 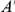 *are alignments of biological sequences and the predictive space* 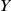 *is equal to* 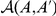 *, that is, the space of the alignments of* 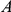 *and* 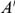 *.*

In the following, 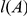 and 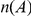 denote the length of the alignment and the number of sequences in the alignment 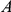, respectively. If both 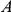 and 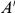 contain a single biological sequence (with no gap), Problem 10 is equivalent to conventional pairwise alignment of biological sequences (Problem 1). As in common secondary structure prediction, the representative estimator plays an important role in this application.

**Estimator 4 (Representative estimator for Problem 10)** *For Problem 10, we obtain the representative estimator (Definition 10). The gain function* 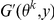 *is the gain function of the* 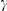 *-centroid estimator. The parameter space* 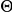 *is represented as a product space* 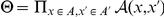 *where* 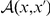 *is defined in the previous section. The probability distribution on the parameter space* 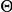 *is given by* 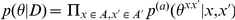 *for* 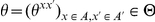 *where* 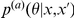 *is given in the previous section (when* 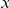 *or* 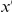 *contains some gaps,* 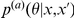 *is defined by the sequences with the gaps removed).*

Corollary 2 proves the following properties of Estimator 5.

**Figure 5:**
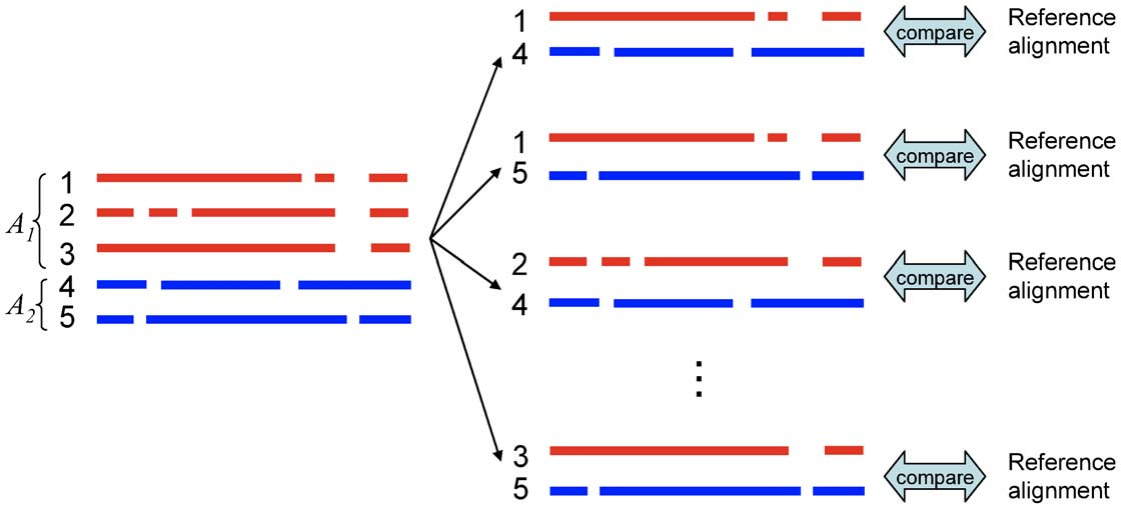
An evaluation process for Problem 10. The comparison between every pairwise alignment and the reference alignment is conducted using TP, TN, FP and FN with respect to the aligned-bases.

**Property 10 (A Relation of Estimator 4 with accuracy measures)** *Estimator 4 is consistent with the accuracy process for Problem 10 that is shown in* *Figure 5* *. We compare every pairwise alignment of* 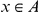 *and* 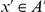 *with the reference alignment. These comparisons are made using TP, TN, FP and FN with respect to the aligned-bases (e.g., using SEN, PPV and F-score).*

**Property 11 (Computation of Estimator 4)** *Estimator 4 can be given by maximizing the sum of probabilities* 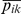 *that are larger than* 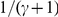 *where*

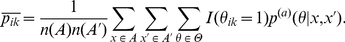
(24)


*Therefore, the pairwise alignment of Estimator 4 can be computed by the Needleman-Wunsch-type DP algorithm of* *Eq. (19)* *in which we replace* 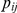 *with* *Eq. (24)* *.*

**Property 12 (Computation of Estimator 4 with** 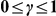 **)** *The Estimator 4 with* 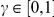 *contains the consensus estimator. Moreover, the consensus estimator is identical to the estimator* 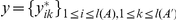 *:*

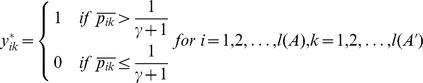

*where* 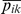 *is defined in* *Eq. (24)* *.*

The probability matrix 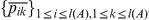 is often called an *averaged* aligned-base (matching) probability matrix of 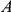 and 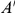. In the iterative refinement of the ProbCons [19] algorithm, the existing multiple alignments are randomly partitioned into two groups and those two multiple alignments are re-aligned. This procedure is equivalent to Problem 10.

The estimator used in ProbCons is identical to Estimator 4 in the limit 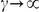. Therefore, the estimator used in ProbCons is a special case of Estimator 4 and it only takes into account the SEN or SPS (sum-of-pairs score) of a predicted alignment.

#### Common secondary structure prediction from a multiple alignment of RNA sequences

**Figure 6:**
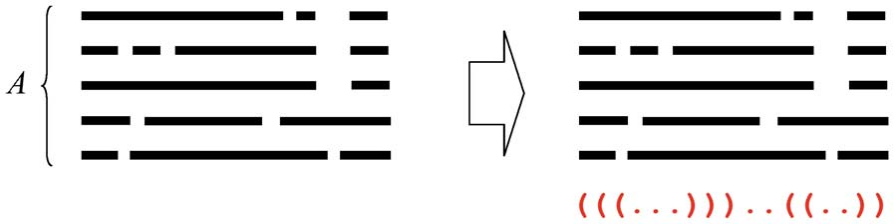
Common secondary structure prediction (Problem 11).

Common secondary structure prediction from a given multiple alignment of RNA sequences plays important role in RNA research including non-coding RNA (ncRNA) [43] and viral RNAs [44], because it is useful for phylogenetic analysis of RNAs [45] and gene finding [43], [ 46]–[48]. In contrast to conventional secondary structure prediction of RNA sequences (Problem 2), the input of common secondary structure prediction is a multiple alignment of RNA sequences and the output is a secondary structure whose length is equal to the length of the input alignment (see Figure 6).

**Problem 11 (Common secondary structure prediction)** *The data is represented as* 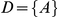 *where* 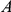 *is a multiple alignment of RNA sequences and the predictive space* 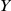 *is identical to* 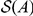 *(the space of secondary structures whose length is equal to the alignment).*

The representative estimator (Definition 10) directly gives an estimator for Problem 11.

**Estimator 5 (The representative estimator for Problem 11)** *For Problem 11, we obtain the representative estimator (Definition 10) as follows. The gain function* 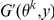 *is the gain function of the* 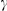 *-centroid estimator. The parameter space is equal to* 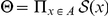 *where* 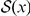 *is the space of secondary structures. The probability distribution on* 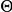 *is given by* 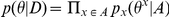 *where* 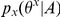 *is the probability distribution of the secondary structures of* 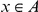 *after observing the alignment* 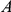 *.*

For example, 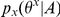 can be given by extending the 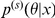, although we have also proposed more appropriate probability distribution (see [49] for the details).

Corollary 2 proves the following properties of Estimator 5.

**Figure 7:**
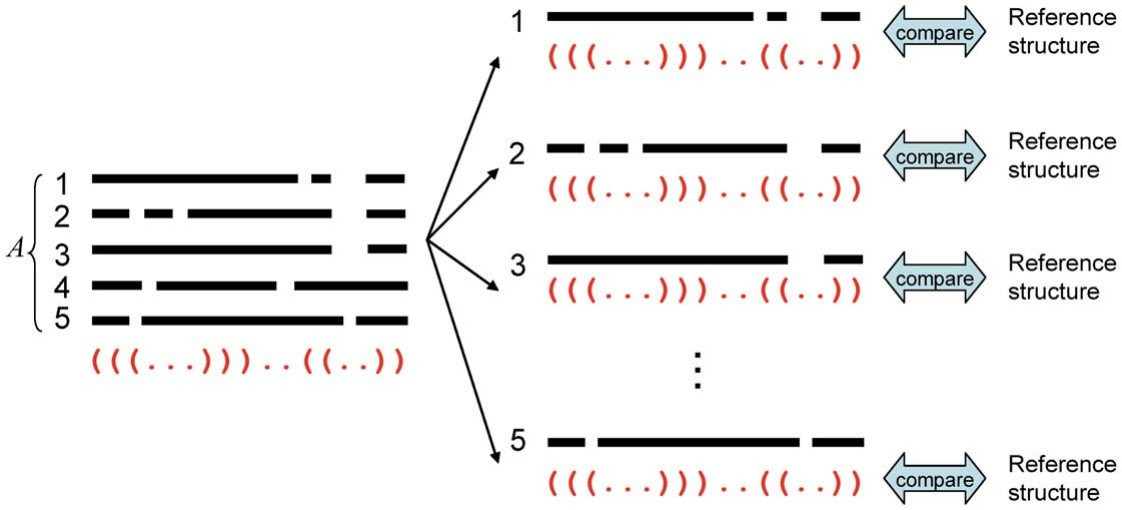
An evaluation process for common secondary structure prediction (Problem 11). The comparison between each secondary structure and the reference secondary structure is done using TP, TN, FP and FN with respect to the base-pairs.

**Property 13 (A relation of Estimator 5 with accuracy measures)** *Estimator 5 is consistent with an evaluation process for common secondary structure prediction: First, we map the predicted common secondary structure into secondary structures in the multiple alignment, and then the mapped structures are compared with the reference secondary structures based on TP, TN, FP and FN of the base-pairs using, for example, SEN, PPV and MCC (* *Figure 7* *).*

Much research into common secondary structure prediction employs the evaluation process in Figure 7 (e.g., [50]).

**Property 14 (Computation of Estimator 5)** *The common secondary structure of Estimator 5 is given by maximizing the sum of the averaged base-pairing probabilities* 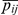 *where*

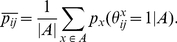
(25)


*Therefore, the common secondary structure of the estimator can be computed using the dynamic programming algorithm in* *Eq. (10)* *if we replace* 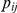 *with* 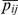 *.*

Also, we can predict the secondary structure of Estimator 5 without conducting Nussinov-style DP:

**Property 15 (Computation of Estimator 5 with** 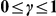 **)** *The secondary structure of Estimator 5 with* 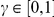 *can be predicted by collecting the base-pairs whose averaged base-paring probabilities are larger than* 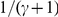 *.*

It should be noted that the tools of common secondary structure prediction, RNAalifold [50], PETfold [8] and McCaskill-MEA [7] are also considered as a representative estimators (Definition 10). In [49], the authors systematically discuss those points. See [49] for details.

#### Pairwise alignment using homologous sequences

**Figure 8:**
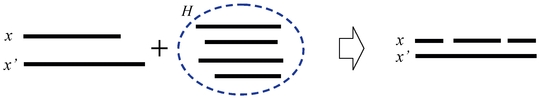
Pairwise alignment using homologous sequences (Problem 12).

As in the previous application to RNA secondary structure prediction using homologous sequences, if we obtain a set of homologous sequences 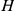 for the target sequences 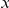 and 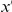 (see Figure 8), we would have more accurate estimator for the pairwise alignment of 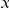 and 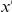 than Estimator 1. The problem is formulated as follows.

**Problem 12 (Pairwise alignment using homologous sequences)** *The data is represented as* 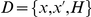 *where* 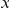 *and* 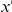 *are two biological sequences that we would like to align, and* 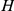 *is a set of homologous sequences for* 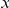 *and* 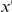 *. The predictive space* 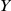 *is given by* 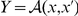 *which is the space of the pairwise alignments of two sequences* 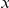 *and* 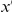 *.*

The difference between Problem 1 and this problem is that we can use other biological sequences (that seem to be homologous to 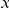 and 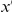) besides the two sequences 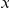 and 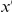 which are being aligned.

We can introduce the probability distribution (denoted by 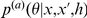) on the space of multiple alignments of three sequences 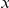, 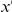 and 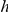 (denoted by 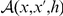 and whose definition is similar to that of 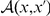) by a model such as the triplet HMM (which is similar to the pair HMM). Then, we obtain a probability distribution on the space of pairwise alignments of 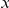 and 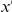 (i.e., 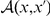) by marginalizing 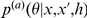 into the space 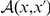:
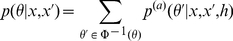
(26)where 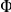 is the projection from 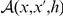 into 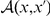. Moreover, by averaging these probability distributions over 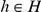, we obtain the following probability distribution on 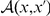:
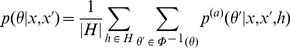
(27)where 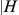 is the number of sequences in 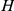.

The 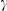-centroid estimator with the distribution in Eq. (27) directly gives an estimator for Problem 12. However, to compute the aligned-base-pairs (matching) probabilities 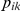 with respect to this distribution demands a lot of computational time, so we employ the approximated 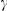-type estimator (Definition 12) of this 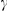-centroid estimator as follows.

**Estimator 6 (Approximated** 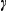 **-type estimator for Problem 12)** *We obtain the approximated* 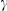 *-type estimator (Definition 12) for Problem 12 with the following settings. The parameter space is given by* 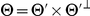 *where*

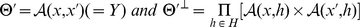

*and the probability distribution on the parameter space* 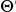 *is defined by *

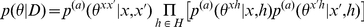
(28)
*for* 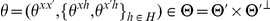 *. The pointwise gain function (see Definition 4) in *Eq. (11)* is defined by *

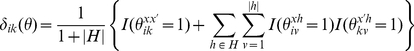
(29)
*where* 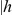 *is the length of the sequence* 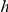 *.*

**Property 16 (Computation of Estimator 6)** *The alignment of Estimator 6 is equal to the alignment that maximizes the sum of* 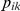 *larger than* 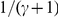 *where*

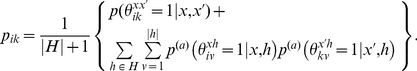
(30)


*Therefore, the recursive equation of the dynamic program to calculate the alignment of Estimator 6 is given by replacing* 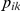 *in* *Eq. (19)* *with* *Eq. (30)* *.*

Moreover, by using Theorem 1, we have the following proposition, which enables us to compute the proposed estimator for 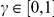 without using (Needleman-Wunsch-type) dynamic programming.

**Property 17 (Computation of Estimator 6 for** 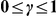 **)** *The pairwise alignment of Estimator 6 with* 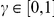 *can be predicted by collecting the aligned-bases whose probability* 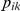 *in (30) is larger than* 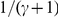 *.*

It should be noted that 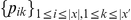 is identical to the *probability consistency transformation* (PCT) of 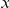 and 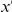 [19]. In ProbCons [19], the pairwise alignment is predicted by the Estimator 6 with sufficiently large 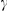. Therefore, the estimator for Problem 12 used in the ProbCons algorithm is a special case of Estimator 6.

#### RNA secondary structure prediction using homologous sequences

**Figure 9:**
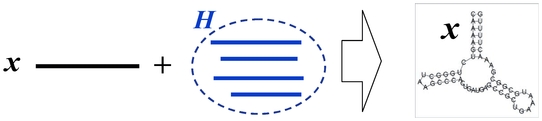
RNA secondary structure prediction using homologous sequences (Problem 13).

If we obtain a set of homologous RNA sequences for the target RNA sequence, we might have a more accurate estimator [23] for secondary structure prediction than the 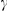-centroid estimator (Estimator 2). This problem is formulated as follows and was considered in [23] for the first time (See Figure 9).

**Problem 13 (RNA secondary structure prediction using homologous sequences)** *The data* 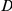 *is represented as* 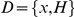 *where* 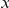 *is the target RNA sequence for which we would like to make secondary structure predictions and* 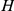 *is the set of its homologous sequences. The predictive space* 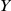 *is identical to* 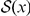 *, the space of the secondary structures of an RNA sequence* 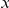 *.*

The difference between this problem and Problem 2 is that we are able to employ homologous sequence information for predicting the secondary structure of the target RNA sequence. In this problem, it is natural that we assume the target sequence 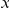 and each homologous sequence 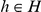 share *common* secondary structures. The common secondary structure is naturally modeled by a *structural* alignment (that considers not only the alignment between bases but also the alignment between base-pairs), and the probability distribution (denoted by 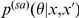) on the space of the structural alignments of two RNA sequences 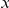 and 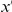 (denoted by 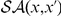) is given by the Sankoff model [51]. By marginalizing the distribution 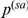 into the space of secondary structures 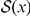 of the target sequence 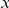, we obtain more reliable distribution 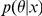 on 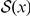: 
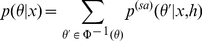
(31)where 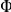 is the projection from 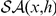 into 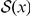. Moreover, by averaging these probability distributions on 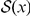, we obtain the following probability distribution of secondary structures of the target sequence. 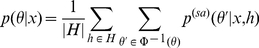
(32)where 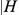 is the number of sequences in 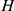. The 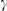-centroid estimator with the probability distribution in Eq. (32) gives a reasonable estimator for Problem 13, because Eq. (32) considers consensus secondary structures between 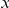 and 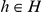. However, the calculation of the 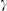-estimator requires huge computational cost because it requires 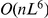 for computing the base-paring probability matrix 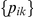 where 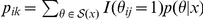 with the distribution of Eq. (32). Therefore, we employ the approximated 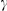-type estimator (Definition 12) of the 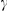-centroid estimator, which is equivalent to the estimator proposed in [23].

**Estimator 7 (Approximated** 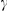 **-type estimator for Problem 13)** *We obtain the approximated* 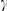 *-type estimator (Definition 12) for Problem 13 with the following settings. The parameter space is given by* 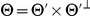 *where*

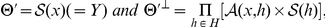

*and the probability distribution on* 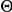 *is defined by *

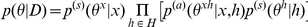

*for* 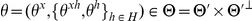 *. Moreover,* *Eq. (11)* *in the pointwise gain function is defined by *

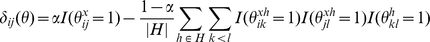

*for* 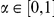 *.*

It should be noted that Estimator 13 is equivalent to the estimator proposed in [23]. The secondary structure of the estimator can be computed by the following method.

**Property 18 (Computation of Estimator 7)** *The secondary structure of Estimator 7 is computed by maximizing the sum of* 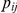 *larger than* 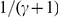 *where*

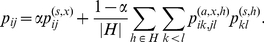
(33)


*Here,* 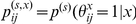 *and* 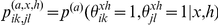 *. Therefore, the secondary structure of Estimator 7 can be computed by the Nussinov-type DP of* *Eq. (10)* *in which we replace* 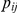 *by* *Eq. (33)* *.*

The computational cost with respect to time for computing the secondary structure of Estimator 7 is 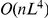 where 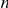 is the number of RNA sequences and 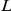 is the length of RNA sequences. In [23], we employed a further approximation of the estimator, and reduced the computational cost to 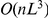. We implemented this estimator in software called CentroidHomfold. See [23] for details of the theory and results of computational experiments. Although the authors did not mention it in their paper [23], the following property holds.

**Property 19 (Computation of Estimator 7 with** 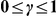 **)** *Estimator 7 with* 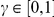 *can be predicted by collecting the aligned-bases where the (pseudo-)base-paring probability of* *Eq. (33)* *is larger than* 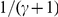 *.*

#### Pairwise alignment of *structured* RNAs

In this section, we focus on the pairwise alignment of structured RNAs. This problem is formulated as Problem 1, so the output of the problem is a usual alignment (contained in 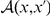). In contrast to the usual alignment problem, we can consider not only nucleotide sequences but also secondary structures in each sequence for the problem. Note that this does *not* mean the structural alignment [51] of RNA sequences, because the structural alignment produces both alignment and the common secondary structure simultaneously.

The probability distributions 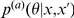 on 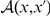 described in the previous section are not able to handle secondary structures of each RNA sequence. In order to obtain a probability distribution on 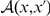 that considers secondary structure, we employ the marginalization of the Sankoff model [51] that gives a probability distribution (denoted by 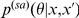) on the space of possible structural alignments between two RNA sequences (denoted by 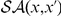). In other words, we obtain a probability distribution on the space 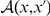 by marginalizing the probability distribution of *structural* alignments of two RNA sequences (given by the Sankoff model) into the space 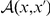 as follows. 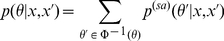
(34)where 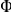 is the projection from 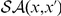 into 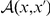, 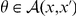 and 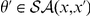. The difference between this marginalized probability distribution and the distributions such as Miyazawa model is that the former considers secondary structures of each sequence (more precisely, the former considers the common secondary structure).

Then, the 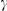-centroid estimator with this distribution Eq. (34) will give a reasonable estimator for the pairwise alignment of two RNA sequences. However, the computation of this estimator demands huge computational cost because it uses the Sankoff model (cf. it requires 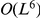 time for computing the matching probability matrix of structural alignments). Therefore, we employed the approximated 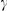-type estimator (Definition 12) of the 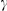-centroid estimator with the marginalized distribution as follows.

**Estimator 8 (Approximated** 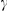 **-type estimator for Problem 1 with two RNA sequences)** *In Problem 1 where* 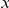 *and* 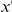 *are RNA sequences, we obtain the approximated* 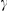 *-type estimator (Estimator 2) with the following settings. The parameter space is given by* 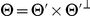 *where*

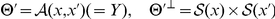

*and the probability distribution on the parameter space* 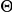 *is defined by *

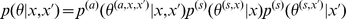

*for* 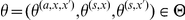 *. The pointwise gain function of* *Eq. (11)* *is defined by *

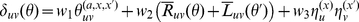

*where*

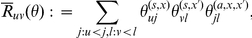


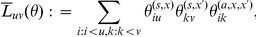


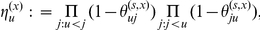

*and* 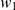 *,* 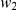 *and* 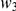 *are positive weights that satisfy* 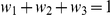 *.*

This approximated 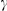-type estimator is equivalent to the estimator proposed in [52] and the alignment of the estimator can be computed by the following property.

**Property 20 (Computation of Estimator 8)** *The alignment of Estimator 8 can be computed by maximizing the sum of probabilities* 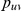 *that are larger than* 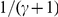 *where*

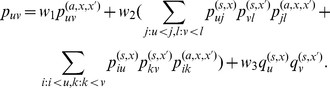
(35)


*Here, we define*

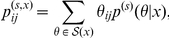


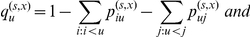


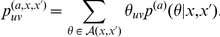



*Therefore, the pairwise alignment of Estimator 8 can be computed by a Needleman-Wunsch-type dynamic program of* *Eq. (19)* *in which we replace* 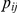 *with* *Eq. (35*)*.*

Note that 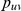 in Eq. (35) is considered as a *pseudo*-aligned base probability where 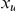 aligns with 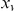.

By checking Eq. (14), we obtain the following property:

**Property 21 (Computation of Estimator 8 with** 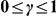 **)** *The pairwise alignment of Estimator 8 can be predicted by collecting aligned-bases where the probability in* *Eq. (35)* *is larger than* 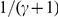 *.*

### Proofs

In this section, we give the proofs of the theorems, propositions and corollary.

#### Proof of Theorem 1

We will prove a more general case of Theorem 1 where the parameter space 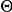 is different from the predictive space 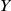 and a probability distribution on 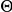 is assumed (cf. Assumption 2).

**Theorem 4** *In Problem 3 with Assumption 1 and a pointwise gain function, suppose that a predictive space* 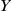 *can be written as*

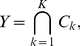
(36)
*where* 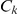 *is defined as*

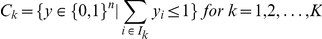

*for an index-set* 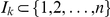 *. If the pointwise gain function in* *Eq. (1)* *(we here think* 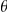 *is in a parameter space* 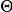 *which might be different from* 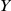 *) satisfies the condition *

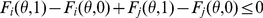
(37)
*for every* 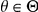 *and every* 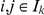 *(* 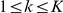 *), then the consensus estimator is in the predictive space* 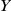 *, and hence the MEG estimator contains the consensus estimator.*

**(proof)** *It is sufficient to show that the consensus estimator* 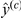 *is contained in the predictive space* 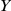 *because* 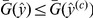 *for all* 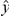 *in the MEG estimators, where*

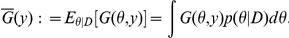



*If we assume that* 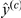 *is not contained in the predictive space,* 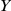 *that is, 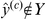, then there exists a* 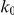 *such that 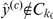. Because* 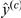 *is a binary vector, there exist indexes* 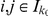 *such that* 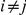 *,* 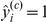 *and* 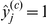 *. By the definition of* 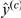 *, we obtain*

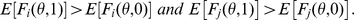



*Therefore, we obtain*

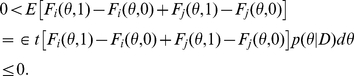



*In order to prove the last inequality, we use Eq. (??). This leads to a contradiction and the theorem is proved.*

**Remark 3** *It should be noted that the above theorem holds for an arbitrary parameter space including continuous-valued spaces*.

**Proof of Theorem 2. (proof)** *Because* 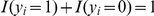 *for arbitrary* 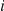 *, we obtain, using the definitions given in* *equations (15* *),(16),(17) and (18),*

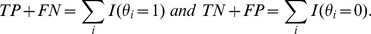



*Therefore, we have *

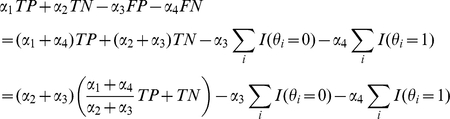

*and this leads to the proof of the theorem.*

**Proof of Theorem 3. (proof)** *The expectation of the gain function of the* 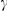 *-centroid estimator is computed as *

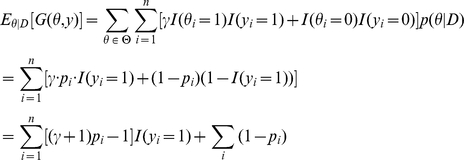

*where* 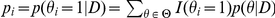 *is the marginalized probability. Therefore, we should always predict* 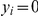 *whenever* 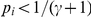, *because the assumption of Theorem 3 ensures that the prediction* 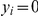 *never violate the condition of the predictive space* 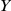 *. Theorem 3 follows by using those facts.*

**Proof of Corollary 1. (proof)** *For every* 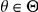 *,* 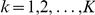 *,* 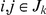 *,* 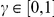 *, we have *

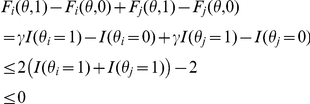

*and the condition of* *Eq. (3)* *in Theorem 1 is satisfied (in order to prove the last inequality, we use* 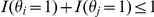 *because* 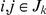 *). Therefore, by Theorem 1, the* 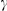 *-centroid estimator contains its consensus estimator.*

*The last half of the corollary is easily proved using the equation *

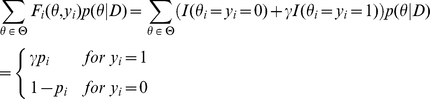

*where* 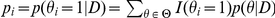 *.*

**Proof of Proposition 1. (proof) 5** *The representative estimator in Definition 10 can be written as*

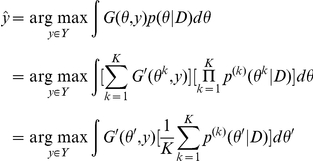



*Then, we finish the proof of Proposition 1.*

#### Derivation of Eq. (14)

The equation is easily derived from the equality 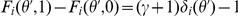.
